# Collaborative care for the detection and management of depression among adults with hypertension in South Africa: study protocol for the PRIME-SA randomised controlled trial

**DOI:** 10.1186/s13063-018-2518-6

**Published:** 2018-03-22

**Authors:** Inge Petersen, Arvin Bhana, Naomi Folb, Graham Thornicroft, Babalwa Zani, One Selohilwe, Ruwayda Petrus, Ntokozo Mntambo, Daniella Georgeu-Pepper, Tasneem Kathree, Crick Lund, Carl Lombard, Max Bachmann, Thomas Gaziano, Naomi Levitt, Lara Fairall, on behalf of the PRIME-SA research team

**Affiliations:** 1 Centre for Rural Health, School of Nursing and Public Health, University of KwaZulu- Natal, Durban, South Africa; 2 Health Systems Research Unit, South African Medical Research Council, Durban, South Africa; 3 Knowledge Translation Unit, University of Cape Town, Cape Town, South Africa; 4 Centre for Global Mental Health, Institute of Psychiatry, Psychology and Neuroscience, Kings College, London, UK; 5 Alan J Flisher Centre for Public Mental Health, Department of Psychiatry and Mental Health, University of Cape Town, Cape Town, South Africa; 6 Biostatistics Unit, South African Medical Research Council, Cape Town, South Africa; 7 School of Public Health and Family Medicine, University of Cape Town, Cape Town, South Africa; 8 Department of Population Health and Primary Care, Norwich Medical School, University of East Anglia, Norwich, UK; 9 Department of Health Policy and Management, Harvard University, Cambridge, USA; 10 Department of Diabetic Medicine and Endocrinology, University of Cape Town, Cape Town, South Africa

**Keywords:** Hypertension, Depression, Primary health care, Low- and middle-income countries, Integrated health care

## Abstract

**Background:**

The high co-morbidity of mental disorders, particularly depression, with non-communicable diseases (NCDs) such as cardiovascular disease (CVD), is concerning given the rising burden of NCDs globally, and the role depression plays in confounding prevention and treatment of NCDs. The objective of this randomised control trial (RCT) is to determine the real-world effectiveness of strengthened depression identification and management on depression outcomes in hypertensive patients attending primary health care (PHC) facilities in South Africa (SA).

**Methods/design:**

The study design is a pragmatic, two-arm, parallel-cluster RCT, the unit of randomisation being the clinics, with outcomes being measured for individual participants. The 20 largest eligible clinics from one district in the North West Province are enrolled in the trial. Equal numbers of hypertensive patients (*n* = 50) identified as having depression using the Patient Health Questionnaire (PHQ-9) are enrolled from each clinic, making up a total of 1000 participants with 500 in each arm. The nurse clinicians in the control facilities receive the standard training in Primary Care 101 (PC101), a clinical decision support tool for integrated chronic care that includes guidelines for hypertension and depression care. Referral pathways available include referrals to PHC physicians, clinical or counselling psychologists and outpatient psychiatric and psychological services. In the intervention clinics, this training is supplemented with strengthened training in the depression components of PC101 as well as training in clinical communication skills for nurse-led chronic care. Referral pathways are strengthened through the introduction of a facility-based behavioural health counsellor, trained to provide structured manualised counselling for depression and adherence counselling for all chronic conditions. The primary outcome is defined as at least 50% reduction in PHQ-9 score measured at 6 months.

**Discussion:**

This trial should provide evidence of the real world effectiveness of strengtheneddepression identification and collaborative management on health outcomes of hypertensive patients withcomorbid depression attending PHC facilities in South Africa.

**Trial registration:**

South African National Clinical Trial Register: SANCTR (http://www.sanctr.gov.za/SAClinicalTrials) (DOH-27-0916-5051). Registered on 9 April 2015. ClinicalTrials.gov: ID: NCT02425124. Registered on 22 April 2015.

**Electronic supplementary material:**

The online version of this article (10.1186/s13063-018-2518-6) contains supplementary material, which is available to authorized users.

## Background

### Burden and significance of co-morbid depression in patients with hypertension

Cardiovascular disease (CVD) (including coronary heart disease (CHD), hypertension and stroke) is the leading cause of mortality in the world [1]. Even in contexts such as South Africa, which has a large human immunodeficiency virus (HIV) burden, CVD is among the top three causes of years of life lost (YLLs) [2]. In relation to disability-adjusted life years (DALYs), which include quantification of YLLs and years lived with disability (YLD), data from South Africa shows a rapid rise in NCDs [2]. Estimates by the World Health Organisation (WHO) using DALYs suggest that NCDs were responsible for 28% of the total burden of disease in South Africa in 2004 [3]. These conditions are becoming more prevalent with the diffusion of urban lifestyle risk factors, including tobacco smoking, unhealthy diet, alcohol consumption and physical inactivity [4–6].

The prevalence of hypertension, at 20% of the adult population, is of particular concern [7], with CVD projected to increase by over 40% in the 35–65 years age group by 2030 [6]. A survey of over 18,000 encounters showed that hypertension follow-up was the leading reason for attending primary care clinics, accounting for 10% of all consultations among adults [8]. Similarly, in a study at a primary care clinic in Khayelitsha, Cape Town, hypertension was the most common morbidity (65%) among 14,364 adult chronic disease patients with tuberculosis (TB), HIV, diabetes or hypertension [9].

NCDs often co-exist with one another, as well as with mental disorders. Results from 245,404 participants from the World Health Surveys in 60 countries revealed that co-morbid depression was present in respondents with diabetes (9.3%), arthritis (10.7%), angina (15%) and asthma (18.1%) [10]. Prevalence rates of depression were highest when there was co-morbidity of two or more chronic physical conditions (23%). Relatively high rates of co-morbidity of NCDs are increasingly being reported in patients in South Africa [11], with a local study also revealing a similar pattern where multiple co-morbidities were associated with increased psychological symptoms [12].

Prevalence of depression in respondents from the World Health Surveys without chronic diseases on the other hand was less than 5% [13]. This suggests that people with NCDs are two to five times more likely to suffer from depression than those without NCDs, with the likelihood increasing with multiple co-morbidities. Depression co-existing with other NCDs was also shown to result in greater health decrements than when one or more physical conditions were present [10]. Mental disorders have a mutually reinforcing relationship with NCDs [14], compromising both prevention and treatment through exacerbating modifiable risk factors, and by compromising adherence and self-care, respectively [15]. In addition, through compromising the endocrine and immune systems, depression and anxiety can have a negative interactive effect at the biological level, exacerbating vulnerability to, and the course of, existing NCDs [16].

With respect to CVD in particular, co-morbid depression increases the risk of CHD and stroke [17–24]. Risk factors for CHD and stroke are related to unhealthy lifestyles which are more common among people with depression or chronic ill-health. Depressed people are more likely to smoke and less likely to exercise, adhere to chronic medication, or eat healthily [25, 26]. It is also possible that there are common causal physiological pathways, such as inflammation, which underlie both CVD and depression [16]. With regard to the impact of common mental disorders on adherence in hypertensive patients specifically, some studies from high-income countries (HICs) show higher non-adherence in patients with co-morbid depression [27]. However, it should be noted that a recent study in Ghana found that there was no relationship between co-morbid depression and/or anxiety and non-adherence, although they did find a relationship between moderate to severe stress symptoms and non-adherence [28].

### Health system reforms to accommodate the rise in co-existing chronic conditions

In response to the rising burden of co-existing chronic diseases that includes HIV, TB and NCDs, the South African National Department of Health has piloted a multi-disease, integrated, chronic services approach [29]. Care for chronic conditions requires collaborative care, which extends beyond the biomedical model that characterises acute care, to incorporate the following elements: self-management support and access to behavioural-change programmes (to help patients have control over their illness and live with their condition); clinical decision support (to improve providers’ expertise and skills to provide the most evidence-informed care); clinical information systems (capturing patient-level information that will help clinical care and follow-up); delivery system design to promote chronic care (promoting planned team-based care and identifying the roles and responsibilities of the different members of the health care team so that they can work synergistically); linkages to community resources such as support groups; and quality improvement to improve the organisation of health care as a whole [30–32].

Such integrated collaborative care for clusters of co-existing illnesses described above, especially physical and mental disorders, has been shown to be more effective than usual care in HICs. Exemplary evidence of this is provided by the TEAMcare trial in the United States which targeted patients with depression and poorly controlled diabetes, heart disease or both, using nurse-led, multi-disease collaborative care [33]. The TEAMcare intervention combined pharmacotherapy with psychosocial interventions, supported by self-help materials, to assist patients to solve problems and set goals to improve adherence and self-care.

In South Africa, at the facility level, the integrated chronic care approach which is part of the integrated clinical management service, aims to strengthen the quality of care for chronic conditions through, inter alia: (1) facility re-organisation to improve the quality of care and servicing all chronic care patients at one service point and (2) strengthening clinical decision support through onsite in-service training using an integrated set of algorithms and checklists developed to support nurse-led identification and management of all common chronic diseases whether communicable, NCDs or mental illness – called PC101 [34]. At the community level, well-controlled patients are supported to engage in self-management by community health worker (CHW)-driven, ward-based outreach teams (WBOTs), which are part of the primary health care (PHC) re-engineering framework in South Africa. At a population level, health promotion and population screening is envisaged to promote an informed and activated population [35].

While mental health problems managed at the PHC level are included in the basket of chronic disease categories catered for by this integrated approach, findings suggest that the limited mental health component included in the basic PC101 training is insufficient to improve PHC nurses’ knowledge of mental health conditions [36], and was not associated with increased case detection of depression in a previous trial focussing on NCDs [37]. There is thus a need to strengthen the mental health component of this integrated approach in order to close the large treatment gap for mental disorders in South Africa, particularly in the chronic care population. While one in six adults experience a common mental disorder (depression, anxiety disorders and substance use disorders) over a 12-month period [38], only one in four receives treatment of any kind [39].

The Programme for improving mental health care (PRIME) [40] in South Africa has been working in one of the districts where this integrated model has been piloted for the past 5 years to develop and evaluate the feasibility of an integrated collaborative-care package for depression and alcohol use disorders (AUD). The package includes strengthening of the mental health component of PC101 training and referral pathways for management of AUD and depression [41]; the use of lay behavioural health counsellors to provide manual-based counselling for chronic care patients with depression, shown to have promising outcomes in a pilot trial [42]; strengthened referral pathways for initiation of psychotropic medication by primary health care physicians; as well as referral to mental health specialists for more severe and treatment resistant depression. In addition, systems strengthening innovations to support these efforts have been introduced through the EMERALD (Emerging Mental Health Systems in Low- and Middle-income Countries (LIMCs)) programme [43], including clinical communication skills for nurse-led chronic care and a strengthened employee assistance programme for primary health care providers experiencing personal problems and burn-out.

Development of the strengthened mental health intervention has been iterative, guided by the United Kingdom (UK) Medical Research Council (MRC) framework for the development of complex interventions [44] and refined during two successive pilots, the first in a single clinic, and the second in a further three clinics. The resultant intervention has been scaled up to 10 clinics in the Dr. Kenneth Kaunda (DKK) District and a further 10 clinics in the adjacent Bojanala Platinum (BP) District during the period 2014–2017. The intervention targets depression co-morbid with all common chronic conditions managed at primary care facilities.

This paper describes one of a pair of trials evaluating the effects of this intervention on mental and physical outcomes among chronic care patients. The other trial (Co-morbid Affective Disorders, HIV and Long-term Health – CobALT), focusses on chronically ill patients receiving antiretroviral (ART) treatment. These two conditions covered by this pair of trials account for most chronic care attendances in South Africa. Similarities and differences between the two trials are described in Table 1. The main distinction is the restriction of the trial reported in this paper to one of these districts, the eligibility criteria for patient participants and the choice of physical outcomes.

**Table 1:** Comparison of the pair of trials evaluating the intervention developed during PRIME-SA

Characteristic	PRIME (PRogramme for Improving Mental health carE-SA) trial	CobALT (Co-morbid Affective Disorders and Long-term Health) trial
Setting	Dr. Kenneth Kaunda District, North West Province, South Africa	Dr. Kenneth Kaunda and Bojanala Districts, North West Province, South Africa
Clinic participants	20 primary care clinics	40 primary care clinics
Patient participants	Patients 18 years or older attending for hypertension treatment with a Patient Health Questionnaire score of 9 or more (*n* = 1000, 50 per clinic)	Patients 18 years or older attending for antiretroviral therapy (ART) with a Patient Health Questionnaire score of 9 or more (*n* = 2000, 50 per clinic)
Number and unit of randomisation	20 primary care clinics	40 primary care clinics
Trial participants	Patients 18 years or older attending for hypertension treatment with a Patient Health Questionnaire score of 9 or more (n = 1000, 50 per clinic)	Patients 18 years or older attending for ART with a Patient Health Questionnaire score of 9 or more (n = 2000, 50 per clinic)
Control arm	The Integrated Services Delivery Model which includes distribution and training in the PC101 guide	Same
Intervention arm	Three additional elements: 1. Clinical communications skills training for nurse clinicians 2. Supplementary training in the mental health components of PC101 3. Clinic-based behaviour-change counsellors equipped to provide morning talks on mental health to promote mental health literacy, manualised counselling for depression (8 sessions, individual or group) and adherence counselling (individual)	Same
Primary mental health outcome	Response at 6 months, defined as a 50% improvement from baseline in the Patient Health Questionnaire 9 score	Same
Primary clinical health outcome	Not applicable	Viral load suppression at 12 months
Duration of fieldwork	April 2015 to December 2016	April 2015 to December 2017
Controlled Trials Registration Number	NCT02425124	NCT02407691
Funding	UK Department for International Development	National Institutes of Mental Health, United States of America

## Objectives

The primary objective of the trial is to determine the real-world effectiveness of strengthened integrated depression care among patients with co-existing hypertension, in a district where the integrated chronic care approach is being piloted. The primary hypothesis of the trial is that patients attending intervention facilities will demonstrate improved depression outcomes, as defined by a 50% reduction in depressive symptoms measured on the Patient Health Questionnaire (PHQ-9) at 6 months post baseline, compared to patients who attend clinics where the strengthened mental health component has not been added to the existing services.

As one of the secondary outcomes, we will determine the impact of the intervention on physical outcomes for hypertension as the trial was under-powered to have this as a primary outcome. We hypothesise that patients attending intervention facilities will demonstrate reductions in their blood pressure at 6 and 12 months post baseline, compared to patients who attend control clinics. Other secondary outcomes for the trial are listed under the ‘Outcome measures’ section.

**Fig. 1:**
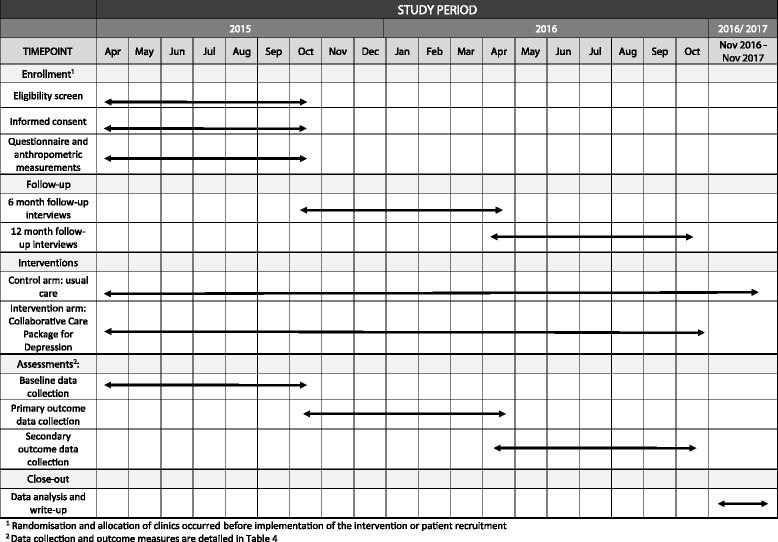
Standard Protocol Items: Recommendations for Interventional Trials (SPIRIT) Figure

Funding and all administrative information required by the Standard Protocol Items: Recommendations for Interventional Trials (SPIRIT) Checklist [45] including the structure, function and composition of all the trial committees, are summarised in the administrative files supplied as Additional files 1 and 2 of the web-based supplementary files (see Additional file 1 for details, Additional file 2 for the SPIRIT Checklist and Fig. 1 for the SPIRIT Figure). The trial is funded from the UK Department of International Development (DFID) through PRIME, supported through grant agreement HRPC10. The funder did not contribute to the study design, data collection and management and will not contribute to data analysis, interpretation, write-up or decisions to submit the results for publication.

## Methods/design

### Trial design

The study design is a pragmatic, two-arm, parallel-cluster randomised controlled trial (RCT) stratified by sub-district. The unit of randomisation is the clinics, and we measure outcomes on individual participants.

### Setting

**Fig. 2:**
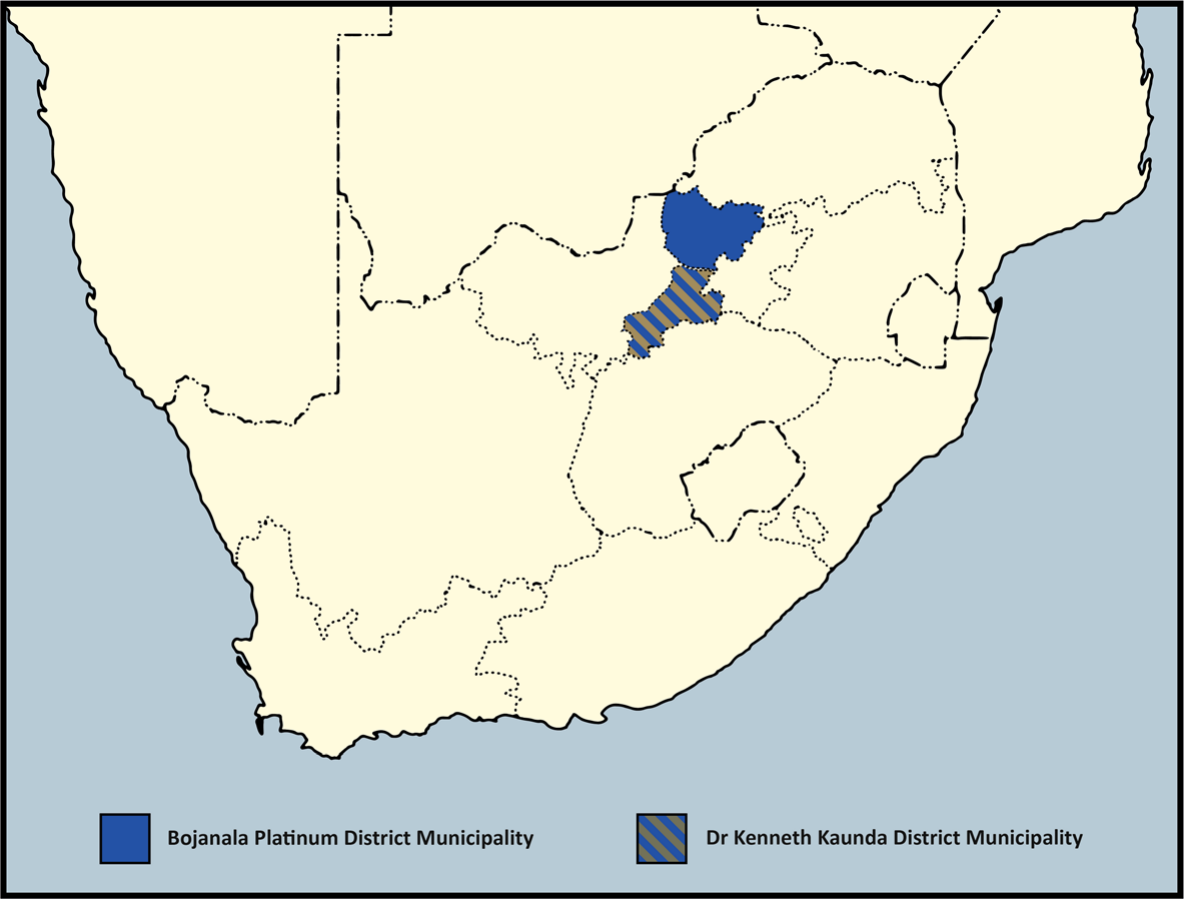
Location of the Dr. Kenneth Kaunda and Bojanala Districts in relation to South Africa

The study site is the DKK District in the North West Province of South Africa located adjacent to and west of the populous Gauteng region (see Fig. 2). The South African National Department of Health guided selection of the site as it is a priority district for several initiatives to strengthen primary care, including the integrated chronic care approach and the establishment of ward-based teams of CHWs. DKK is also one of 11 districts where national health insurance is being phased in over a 14-year period [46]. DKK comprises four sub-districts, with a population of 796,823 [41]. The main economic activities are mining and agriculture. Public health facilities include regional hospitals, primary health care facilities and one specialist in-patient mental health facility.

### Interventions

Control arm: the integrated chronic care approach implemented by the Department of Health (described in the introduction) forms the control comparison and has been implemented in both intervention and control clinics in the year prior to the introduction of the study intervention with various levels of technical support provided by the Department of Health. At facility level the main interventions have been the re-organisation of chronic care services and training of clinic staff in PC101. Queues, appointment systems and waiting rooms for communicable disease services (HIV, TB once the intensive phase of treatment has been completed) have been integrated with services catering for NCDs and mental health, resulting in a single chronic care service aimed at reducing fragmentation and promoting integrated care [47]. Nurses in the control facilities are able to refer patients identified as having depression to PHC physicians for the initiation of antidepressants (as they are not authorised to prescribe antidepressant medications in South Africa); as well as to mental health specialists. The latter are, however, limited with only two district-level PHC psychologists available to service all PHC facilities in the DKK District. Limited specialist psychological and psychiatric care is also available at the district hospital.

**Fig. 3:**
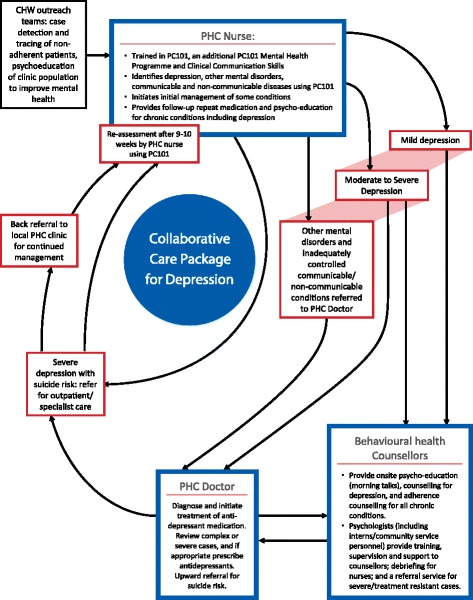
Collaborative-care package for depression

Intervention arm: in the intervention clinics, we strengthened the training of PHC nurse clinicians in the identification and management of depression through training in clinical communication skills and supplementary PC101 mental health sessions (see Additional file 3 of web-based supplementary material for examples of PC101 guidelines). We trained facility trainers in the intervention facilities to deliver this supplementary training material. Facility-based trainers in the control facilities are not exposed to this training in the delivery of this supplementary material. In addition, we strengthened referral pathways for treatment and counselling (see the stepped-up collaborative-care model depicted in Fig. 3). For treatment, we provided supplementary training in mental health care for PHC physicians. For counselling, we introduced lay behavioural health counsellors into the intervention clinics, with structured supervision from the project psychologist and district-based psychologists that includes individual and weekly group supervision. A comparison of the training provided in the control and intervention clinics is contained in Table 2. The intervention coordinator is responsible for monitoring the intervention to ensure that each component is functioning optimally at the facility level. A detailed monthly monitoring document was developed and is used to monitor coverage of nurse training in the supplementary material given high nurse turnover, nurse referrals to counsellors and counsellor integration within the facility.

**Table 2:** Comparison of training provided to control and intervention clinics

Provider	Role	Training	Content of training	Method and timeframe
Control and intervention facilities				
PHC nurses	Identifies, provides brief interventions and refers	Basic onsite PC101 training	Case scenarios used for training in the identification and management of common chronic diseases, including communicable diseases, NCDs (including hypertension), women’s health and mental health. Mental health components draw on the WHO’s mhGAP guidelines [67] and adopt a syndromic approach to mental health symptoms (such as stress, insomnia, suicidal thinking) with diagnostic algorithms and treatment checklists for depression	(1) PC101 master trainers train facility trainers who train PHC nurses at the facilities
				(2) 12 weekly sessions over 12 weeks at facilities (2 of which are on mental disorders)
				(3) Training uses case-scenario material of patients with chronic conditions, including co-morbid conditions
Intervention facilities				
PHC nurses	Identifies, provides brief interventions and refers	Orientation and clinical communication skills training	(1) Overview of the system changes being made by the DoH in South Africa to accommodate the demands of integrated chronic care; their role as case managers within the collaborative-care model for depression	4 2-h interactive workshops at PHC facilities/regional training centre
			(2) Orientation to patient-centred care and clinical communication skills necessary to implement patient-centred care	
			(3) Skills to manage patient emotions within the consultation; self-care including how to cope with their own emotions and burn-out	
			(4) Motivational interviewing skills to promote patient self-management	
		PC101 supplementary training in mental health	(1) Detection of depression and anxiety, psychoeducation and referral to counsellors and/or physician for consideration of psychotropic medication in the case of moderate to severe depression	(1) PC101 master trainers train facility trainers (2-day workshop) who train PHC nurses at the facilities
			(2) Detection of risky alcohol use and brief intervention for harmful/hazardous drinking and for detoxification and referral to specialist rehabilitation programmes for dependency as per the mhGAP guidelines [68]	(2) 3 weekly sessions over 3 weeks at facilities, with an additional follow-up session 1 month later
			(3) Assessment of suicide intent	
			(4) Patient review after 8 weeks to assess response to treatment and onward referral for specialist care as indicated by the mhGAP evidence-based guidelines for LMICs [68] if necessary following a treatment-to-target approach as contained in the collaborative-care model (see Fig. 3). Treatment to target involves tracking a patient’s symptom severity and adjusting or intensifying treatment should patients not show an improvement in symptoms following initial treatment [69]	(3) Training uses case scenarios case scenario material of chronic patients with co-morbid mental disorders
PHC physicians	Diagnoses, initiates and monitors response to psychotropic medication	Orientation and training in mhGAP/PC101	(1) Orientation to the importance of treating co-morbid depression	3 1-day workshops spread over 6 months
			(2) Training in mhGAP guidelines	
			(3) Follow-up using case studies of patients	
Behavioural health counsellors	Provides evidence-based counselling	Counselling training	(1) Manualised counselling package comprising 8 sessions (delivered individually or in groups)	1 week of off-site training; 1 week of peer-to-peer mentoring; in-vivo supervision by a psychologist of each session; weekly follow-up group supervisory sessions, augmented where possible by weekly individual supervision sessions
			(2) Session 1: psychoeducation session on depression; the last session is a closure session; sessions 2-7 draw on problem solving and cognitive behavioural techniques, including behavioural activation to address the common triggers of depression and anxiety which, in this population, include poverty, interpersonal conflict, social isolation and avoidance, grief and loss, and stigma that emerged from qualitative interviews held with service users with depression during the formative phase of the PRIME project in South Africa in 2 provinces [70]. A prototype had been field tested in KwaZulu-Natal and positive results demonstrated in an individually randomised pilot trial [42, 71]; adherence session provides information on the chronic condition/s and chronic medication/s the patients may have as well as helping patients with adherence difficulties	
			(3) While developed to treat depression, the intervention has been found to promote improvements in global psychological functioning as well [42, 71], thus having the potential for trans-diagnostic effects, in line with evidence that diagnosis-specific cognitive-behavioural therapy has beneficial effects on untargeted co-morbid emotional disorders [72]	
Specialists (psychologist/psychiatrist)	Training, supervision of counsellors	Orientation to task sharing	Psychologists (including interns and community service psychologists) orientated to their roles	One-off workshops

*NCD* noncommunicable diseases, *PHC* primary health care, *WHO* World Health Organisation

### Clinic participants

We considered all 60 public sector primary care clinics in the DKK District for enrolment in the trial and excluded clinics if they were mobile or satellite units, saw fewer than 10,000 attendances per year, or participated in the piloting of the intervention or data collection. We enrolled the largest 20 eligible clinics in the trial.

### Randomisation, blinding and allocation concealment

We randomised clinics, their staff and patients to one of two parallel groups, with equal numbers of clinics (*n* = 10) in each group, arranging clinics into two strata determined by their sub-district to avoid potential confounding resulting from geographically determined differences in sub-district management. Twelve clinics serving the central Matlosana sub-district comprised one strata, and eight more rurally situated clinics from the Tlokwe, Maquassi Hills and Ventersdorp sub-districts the second stratum. We randomised clinics in each stratum on a 1:1 ratio yielding 10 clinics per group. Randomisation was completed using nQuery Advisor by the trial statistician independently of the managers who gave permission for the trial, and before implementation of the intervention or patient recruitment to ensure allocation concealment.

Intervention clinic staff cannot be blinded to the clinic’s treatment status given that they have to agree to additional training activities over and above the standard PC101 training; as well as the introduction of behavioural health counsellors. Although the outcome assessors (the fieldworkers) are not informed as to which clinics are in the intervention arm and which are in the control arm, it is not possible to guarantee complete blinding of the fieldworkers given the abovementioned intervention activities at the intervention clinics. To minimise the potential for bias, all patient screening, recruitment, interviewing, follow-up and quality assurance procedures are standardised across both arms to ensure that these data collection activities and procedures are applied equally in all clinics. The data collection team also operates independently of the intervention team, so as to limit exposure to the intervention.

### Patient participants

**Fig. 4:**
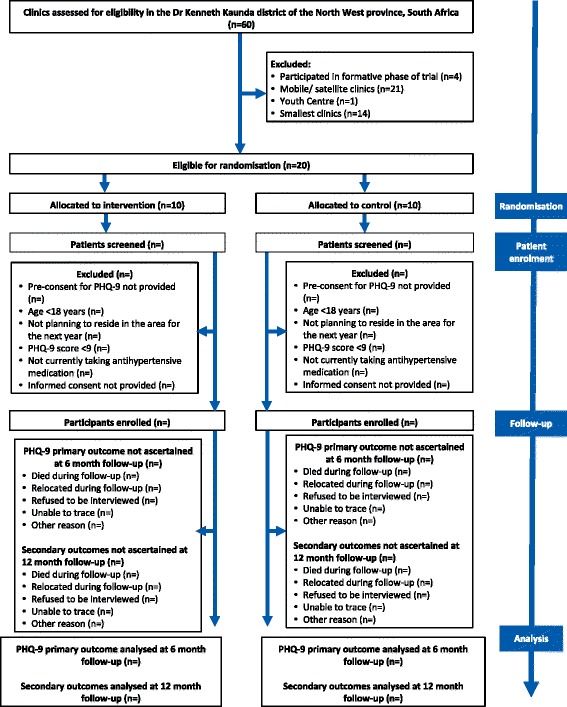
Flow of participants

We recruit patient participants from the ambulatory primary care population attending chronic care services at the trial clinics. Participants are considered to be eligible for the trial if they are aged 18 years or older, report receiving treatment for hypertension and screen positive for depression with a total score of 9 or more on the (PHQ-9) [48] (see Fig. 4). We chose the PHQ-9 given that it has been commonly used in other trials on depression [42, 49–51], and is relatively short and easy to train fieldworkers to administer. Most importantly, it has also been validated on the target population in South Africa [52]. We did consider other depression measures such as the Hamilton Rating Scale for Depression (HAM-D). The HAM-D, however, can take up to 15 to 30 min to administer, is ideally clinician administered and is also complicated to score, requiring substantial training in getting reasonable inter-rater agreement [53]. Given that this is a pragmatic trial, our preference was to use a measure that could be relatively easier to administer and which had been validated in the population of interest. The PHQ-9 is aligned with the *Diagnostic and Statistical Manual of Mental Disorders-Text Revision* (DSM-IV-TR) diagnostic criteria for major depressive disorder and the cut-off of 9 has been determined for the study population in a preliminary validation study involving 676 chronic care patients also from the DKK District. A cut-off of 9 and above was shown through the validation study to be the optimal cut-off for sensitivity and specificity indices against the ‘gold standard’ of a Structured Clinical Interview for a DSM-IV (SCID) diagnosis of major depression [51]. The trials findings will, therefore, apply to patients with major depressive symptoms at baseline. In this validation study, performance of a Setswana translation of the PHQ-9 with an adapted response to improve understanding of the time period when symptoms were experienced was compared against a structured clinical interview administered by a clinical psychologist [52] (see Table 3 for a comparison of the original and localised validated version). Mean PHQ-9 scores at baseline will be compared between intervention and control arms to assess for any difference which could indicate potential selection bias. We exclude participants if they are in need of urgent medical attention, too ill to provide informed consent or plan to move outside the vicinity of the clinic during the 12 month follow-up period (see Fig. 4). We expect some patients to meet eligibility criteria for both pairs of trials owing to the growing overlap between HIV and hypertension, recognise that it is important to represent this co-morbidity within the trials, and so invite such patients to participate in both trials.

**Table 3:** Patient Health Questionnaire-9: general and localised versions

Over the last 2 weeks, how often have you been bothered by any of the following problems?^a^	Not at all	Several days	More than half of the days	Nearly every day
Over the last 2 weeks, how often have you been bothered by any of the following problems?^b^	0 days	1–7 days	8–11 days	12–14 days
Little interest or pleasure in doing things	0	1	2	3
Feeling down, depressed, or hopeless	0	1	2	3
Trouble falling or staying asleep, or sleeping too much	0	1	2	3
Feeling tired or having little energy	0	1	2	3
Poor appetite or overeating	0	1	2	3
Feeling bad about yourself – or that you are a failure or have let yourself or your family down	0	1	2	3
Trouble concentrating on things, such as reading the newspaper or watching television	0	1	2	3
Moving or speaking so slowly that other people could have noticed? Or the opposite - being so fidgety or restless that you have been moving around a lot more than usual	0	1	2	3
Thoughts that you would be better off dead or of hurting yourself in some way	0	1	2	3
Please could you confirm your answer for this question: Over the last 2 weeks, how often have you been bothered by thoughts that you would be better off dead or of hurting yourself in some way	0	1	2	3
	Total score ___ = ___ + ___ + ___			
If you checked off any problems, how difficult have these problems made it for you to do your work, take care of things at home, or get along with other people?^a, b^	Not difficult at all	Somewhat difficult	Very difficult	Extremely difficult

^a^General version

^b^Localised version

We will enrol roughly equal numbers of depressed hypertensive patients into the study using the PHQ-9, across the 20 clinics with a target of 50 participants per clinic, making up a total of over 1000 participants with over 500 in each arm (see Fig. 4).

### Patient screening and recruitment

In the intervention clinics, we recruit patients following training of nurses and counsellors in the intervention as well as a 2-month embedding period. Trained fieldworkers enrol participants in the study independent of clinical care or participation in the counselling intervention for depression. Screening follows a three-stage process:

1. Trained fieldworkers (recruiters) will explain the study to patients seated in the chronic care clinic waiting room and invite them to participate. No mention of depression or hypertension is made at this stage to avoid possible stigma being attached to patients who identify themselves as interested. Fieldworkers then ask these patients one-on-one whether they are receiving hypertension treatment and if they plan to reside in the area for the next 12 months.
2. Fieldworkers then escort patients who respond positively to both these questions to another fieldworker, trained to perform the interview, located in a private space in the clinic. The interviewers brief potential participants about the psychological content of the questions in the PHQ-9 and obtain verbal consent before continuing (this pre-consent process is contained in Additional file 4 of web-based supplementary files). Patients who score 9 or more on the PHQ-9 are then taken through the detailed information sheet and asked to provide written informed consent (see Additional file 5 of the web-based supplementary files for more detail). We collect patient identifiers only once written consent is provided. We consider this opt-out verbal consent process necessary to balance the risks associated with the emotional content of the screening questions and the practicalities of administering written consent to many thousands of patients, as preliminary work in the district indicated that we could expect around 12% of patients to screen positive for depression using the PHQ-9.
3. We collect participant identifiers only once written consent is provided, and request national identity numbers to assist linkage with the national population register, which records deaths in SA.
  We ask illiterate participants to confirm that they have understood each consent line and mark a cross (x) in each consent line which an independent witness then witnesses and countersigns.

If patients show signs of suicidality through a positive response to the suicidal question of the PHQ-9, we repeat the question to confirm the response. We give patients who report having suicidal thoughts in the past 2 weeks psychoeducational material on suicidal thoughts and contact information about where to get help. We refer patients with suicidal thoughts on more than 7 days in the past 2 weeks to the nurse on the same day for assessment and onward referral if necessary.

### Data collection and outcome measures

Participants provide written consent before completing the full baseline questionnaire in the language of their choice, predominantly Setswana although some interviews are also conducted in Afrikaans or English. This questionnaire includes items on demographics and multiple instruments (outlined below, Table 4) (see Additional file 6 of supplementary files for full questionnaire). Anthropometric measurements include height, weight, waist circumference and blood pressure. Trained fieldworkers measure the latter using a digital sphygmomanometer, and take readings while the participant is seated at three time points during the course of the interview. We use the ‘Averaging Rules for Determining Mean Blood Pressure’ from the *National Health and Nutrition Examination Survey (NHANES) Health Technology/Blood Pressure Procedures Manual* [54] to determine the average blood pressure whereby the first measurement is discarded and the second two readings used to calculate a mean blood pressure for each assessment (baseline, 6 months, 12 months).

**Table 4:** Schema of patient-level data collection

Outcome						
				Baseline	6 months	12 months
Outcome	Measurement	Source	Metric			
Primary measurements						
Depression symptoms	PHQ-9	Self-reported	50% reduction in PHQ-9 score	●^a^	●●^b^	●
Secondary mental health outcomes						
Depression symptoms	PHQ-9	Self- reported	50% reduction in PHQ-9 score	●	●	●●
Depression symptoms	PHQ-9	Self- reported	Remission defined as score of < 5 on PHQ-9	●	●	●●
Depression symptoms	PHQ-9	Self- reported	Mean PHQ-9 scores at 6 and 12 months	●	●●	●●
Antidepressant treatment		Self- reported	Proportion with antidepressant treatment initiated or intensified	●	●	●●
Counselling		Self- reported	Proportion receiving counselling by clinic-based counsellor	●	●	●●
Referral to specialist mental health worker/service		Self- reported	Proportion referred	●	●	●●
Stress	Perceived Stress Scale	Self-reported	Mean score	●		●●
Secondary hypertensive outcomes						
Blood pressure		Interviewer measured	Difference in means	●	●●	●●
Retention in care		Self-reported; clinic records	Proportion in care	●		●●
Integrated care outcomes						
Cardiovascular risk factors	Blood pressure, weight, Body Mass Index, waist circumference	Interviewer measured	Difference in means	●	●	●●
Diagnosis of other co-morbid illnesses		Self-reported	Proportion diagnosed			●●
Quality of chronic illness care received	Patient Assessment of Care for Chronic Conditions (PACIC)	Self-reported	Mean PACIC score	●		●●
Health economic outcomes						
Health care utilisation		Self- reported; linkage with hospitalisation databases	Incidence rate ratio	●		●●
Productivity and economic outcomes		Self- reported		●		●●
Disability	WHO Disability Assessment Schedule 2.0	Self- reported	Mean score	●		●●
Safety measurements						
Hospitalisation		Self- reported; linkage with hospitalisation databases	Proportion hospitalised	●	●	●●
All-cause mortality		Clinic, report, linkage with mortality register	Proportion who died	●	●	●●
Suicide		Follow-up of cause of all known deaths with clinic and family interview	Proportion of suicides	●	●	●●

^a^● data measured

^b^●● time when endpoint for outcome will be reported

The primary outcome measure is a reduction in PHQ-9 score of 50% or more compared with baseline [49–51, 55, 56], evaluated at 6 months. Thus, we use the PHQ-9 to determine eligibility for the trial as well as to measure mental health outcomes at 6 and 12 months.

Secondary outcomes (see Table 4) include: (1) response at 12 months defined as a 50% reduction in the score on the PHQ-9, (2) remission at 12 months defined as a score of < 5 on the PHQ-9, (3) mean PHQ-9 scores at 6 and 12 months, (4) antidepressant treatment, including initiation or intensification of antidepressant medication, (5) referral to a counsellor for depression counselling, (6) referral to a mental health specialist (clinical psychologist, psychiatrist or secondary care services), (7) blood pressure at 6 and 12 months, (8) disability measured at 12 months using the WHO Disability Assessment Schedule version 2.0 (WHODAS 2), (9) stress symptoms measured at 12 months using the Perceived Stress Scale (PSS), (10) patient assessment of quality of chronic illness care received measured at 12 months using the Patient Assessment of Care for Chronic Conditions (PACIC) scale, (11) health care utilisation including clinic visits and hospital admissions, (12) resource use and economic outcomes measured using a Service Use Questionnaire and (13) all-cause mortality measured through follow-up at the clinic and linkage to the South African population register.

We obtain *demographic information* through self-report at baseline, including questions on sex, age, educational and employment status, income and household composition. We repeat some of the questions at 6- and 12-month interviews to track changes in economic status.

The *WHODAS 2* [57] is a generic assessment instrument for disability for diseases including mental disorders. We use the 12-item interviewer administered version which has been previously used on a South African ART patient population [58].

The *Perceived Stress Measure (PSS)* [59] is a 10-item self-reported measure that provides an assessment of the extent to which situations in a person’s life are appraised as stressful. It has been previously used in South Africa with perceptions of high levels of stress associated with higher risk for developing some mental disorders [60].

The *Patient Assessment of Care for Chronic Conditions (PACIC) scale* is a self-reported measure of quality of patient-centred chronic clinical services received in the past 6 months, which emphasises self-management support in the form of collaborative goal setting, problem-solving and follow-up [61]. The original 20-item scale was adapted for the local context and reduced to 10 items.

### Limitations

Limitations of the trial design include dependence on self-reporting of hypertensive medication for inclusion in the trial, lack of detailed information on prescribed medication for hypertension, and limited data on adherence to medication. While the broad inclusion criteria and light-touch follow-up are consistent with the pragmatic design and are intended to maximise generalisability to an intervention in real-world circumstances, it can be difficult to assess whether all the study participants were eligible for the intervention and to quantify the processes of care in a causal pathway. A qualitative process evaluation will be conducted alongside the RCT to address these gaps and to understand how integrating mental health services for depression affects care of physical conditions like hypertension.

### Data management

Fieldworkers use handheld devices to collect data that is uploaded onto a secure server. The Trial Management Team manage data quality assurance through weekly telephonic data meetings when uploaded data is reviewed, as well as bimonthly face-to-face meetings of the Data Management Team comprising the data manager, principal investigators (PIs) and invited co-investigators, including the statistician depending on the need. Data is collected alongside the first phase of the CobALT trial in the DKK District, and is subject to the same standard operating procedures for data management for the CobALT trial. These include intensive fieldworker training in the administration of the interview, and onsite supervision by three fieldwork supervisors who perform daily quality checks on interviews conducted, observe interviews weekly to ensure the interviewer administers the questionnaire and performs the measurements as trained, and assists in addressing fieldwork problems that may have impeded data collection.

Patients receive a ZAR50 voucher (just under US$3) in appreciation for their time. We schedule follow-up appointments for 6 and 12 months at the clinic, with reminders and confirmation of follow-up interviews provided using text messaging and, where appropriate, telephone calls.

#### Sample size and power calculations

We calculated sample size on the following assumptions: we considered the primary outcome as the proportion of respondents considered to have at least a 50% reduction in PHQ-9 scores at 6 months compared with baseline; an intraclass correlation coefficient (ICC) of 0.04 for this outcome and is based on a similar trial in a LIMC setting [62], a significance level of 0.05; and loss to follow-up of 15% to make provision for patients who may leave the area, die or refuse to take part in the study at follow-up. This loss-to-follow-up rate was determined on the basis of a previous trial in South Africa involving chronic care patients, including those with depression [63]. The trial statistician calculated a sample size comprising 50 patients in each of the 20 facilities making up 1000 patients (500 patients in the intervention arm and 500 patients in the control arm) to provide 90% power to detect an effect size of 17% (60% control versus 77% intervention), and 80% power to detect an effect size of 15% (60% control versus 75% intervention).

#### Statistical methods

We will analyse data using the STATA statistical package and will blind all analysis pertaining to the primary and secondary objectives until the analysis is finalised. At baseline, we will calculate descriptive statistics including frequencies, means and standard deviations on both the intervention and control arms to establish comparability of the arms. For the intention-to-treat analysis of the primary outcome, we will use binomial regression to estimate differences in proportions of participants considered having at least 50% reduction in depressive symptoms, as measured using PHQ-9 at 6 months. We will take into account the clustering of participants within facilities for any inference done. We will complete secondary analyses whereby we adjust for baseline PHQ-9 scores, and will test for interaction effects between physical conditions. Secondary analysis will also include a comparison of outcomes in intervention-group participants who did and did not receive the intervention with adjustment for baseline covariates; as well as sub-group analysis of blood pressure outcomes in patients who had uncontrolled blood pressure at baseline.

#### Process evaluation

The UK MRC framework for the process evaluation of complex evaluations [64] informs the process evaluation design. We have adopted a mixed-methods approach that combines quantitative process variables collected during the 12-month period of the trial and post hoc in-depth qualitative process evaluation interviews across the clusters that follow analysis of the trial outcome data. Through monitoring changes at a facility and district level on a regular basis, we will gather information related to contextual factors that may influence outcomes in the intervention and control clusters. To monitor fidelity and quality of implementation we will collect (1) process indicators on coverage of training sessions and supervision received across the intervention clusters for PHC providers and behavioural health counsellors, (2) data on the total number of patients identified and referred for treatment of depression, and (3) fidelity checks of the counselling implementation through analysing audio-recordings of counselling sessions. We will employ in-depth qualitative process evaluation interviews to examine variations in the trial outcomes associated with potential causal mechanisms and contextual factors. These interviews will be informed by the 6-month depression primary outcome data, and conducted *post hoc* on purposefully sampled clusters and individuals to understand variations in the clusters that may emerge in the outcome data and analysed using thematic analysis [65].

#### Ethical considerations

In order to protect the confidentiality of the information provided by research participants, the research team, including fieldworkers, are required to sign a confidentiality agreement. The hand-held data collection devices are also password protected and data from interviews are uploaded immediately onto a secure server which is permission and access controlled, at which point they are erased from the hand-held device. Access to the uploaded data is restricted to the PIs, data manager, data architect, site project coordinator and research manager. All data collected will also be anonymised for analysis purposes.

#### Monitoring of adverse events

The Data and Safety Monitoring Board (DSMB) for the COBALT trial also provides safety monitoring for the PRIME-SA trial. Specific adverse events, applicable to the PRIME-SA trial, and protocols for management and reporting are specified in a DSMB Charter and are detailed in Table 5. Of note, given the high rate of positive responses to the ninth item of the PHQ-9 (‘Thoughts that you would be better off dead or of hurting yourself in some way’) during the past 2 weeks in the formative PRIME work, and a low rate of reported deaths by suicide in the North West Province, the DSMB advised against over-interpretation of a single item in a questionnaire. We thus introduced a standard operating procedure previously described that distinguishes patients needing psychoeducation, where patients are provided with information on their condition and where to get help from local community resources and helplines, from those requiring immediate assessment by the clinic nurse and onward referral. Death is identified during the follow-up periods and the cause of death investigated by the fieldworkers through contacting the family and through reviewing the clinic files and the National Death Registry. Death by suicide is considered a serious adverse event as it could be associated with the intervention and is reported to the COBALT DSMB and UKZN BREC within 7 days of knowledge of confirmed suicide.

**Table 5:** Defining, monitoring and reporting of harm in the PRIME trial

Type of harm	Source and method of identification	Action(s) to mitigate harm to specific participants	Reporting frequency and to whom
Adverse events			
Positive response to ninth item of the PHQ-9: ‘Thoughts that you would be better off dead or of hurting yourself in some way’	Participant interviews (baseline, 6 month follow-up, 12 month follow-up). Flag within electronic questionnaire prompting interviewer to act	Repeat question to reduce telescoping-type reporting errors. If ≥ 8 days in last 2 weeks, immediate referral to clinic staff. If between 1 and 7 days then written educational material given	6-monthly report to DSMB 6-monthly to IRB (with DSMB letter of recommendation)
PHQ-9 score of ≥ 20 at 12 months suggesting persistent severe depression	Participant interviews (12-month follow-up). Data report (monthly)	Summary forwarded to clinic together with recommendations for further treatment	6-monthly report to DSMB 6-monthly to IRB (with DSMB letter of recommendation)
Blood pressure severely raised (≥ 180/110) placing participant at immediate risk of cardiovascular event	Participant interviews (baseline, 6-month follow-up, 12-month follow-up). Flag within electronic questionnaire prompting interviewer to act	Immediate referral to clinic staff for review	6-monthly report to DSMB 6-monthly to IRB (with DSMB letter of recommendation)
Raised blood pressure at follow-up representing undiagnosed or uncontrolled hypertension	Participant interviews (baseline, 6-month follow-up, 12-month follow-up). Longitudinal patient record	Summary forwarded to clinic together with recommendations for further treatment	6-monthly report to DSMB 6-monthly to IRB (with DSMB letter of recommendation)
Serious adverse events			
Hospitalisation	Participant interviews (baseline, 6-month follow-up, 12-month follow-up). Routinely collected hospitalisation data. Data report (monthly).	No immediate action other than 6-monthly review by DSMB	6-monthly report to DSMB 6-monthly to IRB (with DSMB letter of recommendation)
Death (excluding suicide)	Participant interviews (Loss to Follow-up Form). National Population Register. Data report (monthly)	No immediate action other than 6-monthly review by DSMB	6-monthly report to DSMB 6-monthly to IRB (with DSMB letter of recommendation)
Death by suicide	Participant interviews (Loss to Follow-up Form). National Population Register (providing we are able to access cause of death). Data report (weekly)	Immediate notification of PI (LF) who will follow-up with fieldwork staff to confirm suicide and establish date of suicide	Notification of IRB, DSMB and NIMH within 7 days of knowledge of confirmed suicide

*DSMB* Data and Safety Monitoring Board, *IRB* Institutional Review Board, *PHQ-9* Patient Health Questionnaire-9, *PI* principal investigator

Further, while hospitalisation, prolonged hospitalisation and death would normally be considered serious adverse events, these events are expected to occur fairly frequently among the participant population given that they all suffer from hypertension which may also be co-morbid with other chronic conditions, thus increasing their vulnerability to hospitalisation and premature death. We thus monitor hospitalisations and deaths at 6- and 12-month follow-up interviews as well as link hospitalisation data on a quarterly basis, with a once-off linkage with the national population register being planned as well. The frequency of these events are reviewed at the 6-monthly DSMB meetings, which are then reported to the relevant Institutional Review Board (UKZN) with the DSMB Letter of Recommendation.

### Dissemination

The PIs have established a Trial Steering Committee comprising key national, provincial and district stakeholders including policy-makers, planners and managers (see Additional file 1 supplementary web-based material for more detail) where trial progress is being reported and provides a platform to disseminate the trial results and partake in discussions around policy implications. These stakeholders have expressed their commitment to implementing the intervention package should the trial results be successful. Scale-up would require, inter alia, the expansion of the PC101 training to include clinical communication skills; a dedicated mental health module; and role clarification of the lay behavioural health counsellors within the collaborative-care model.

Further, the results of the trial will be written up in a main outcome publication within a year of the finalisation of the fieldwork, together with additional papers detailing secondary outcomes and sub-group analyses. Authorship will be determined on the basis of contributions made to the design, intervention, data collection and analysis.

## Discussion

In the context of the rising burden of NCDs in LMICs, and CVD specifically in South Africa, the high prevalence of co-morbid depression is a major and growing public health threat. It interferes with treatment adherence as well as health-enhancing lifestyle changes, and has the potential to undermine investments in health care for these conditions. There is an urgent need to close the treatment gap for common mental disorders in LMICs and South Africa specifically, especially in patients who have multiple co-morbid chronic conditions – given the negative health impacts and economic costs. It is particularly important to know whether such an intervention leads to cost saving both to the health care system (through improved physical health outcomes and reduced health care utilisation) and broader society (through improved functioning, productivity and income).

Given the paucity of mental health specialists in LMICs, task sharing has been recommended as a strategy to close this gap globally as well as locally in South Africa [66, 67]. PRIME in South Africa, in collaboration with the Department of Health has developed a collaborative-team-based package for depression care in the chronic care population that strengthens and builds on existing task-sharing strategies. The need for this RCT to evaluate the real-world effectiveness of this integrated collaborative approach is spurred by the lack of evidence on the effectiveness of collaborative-care models on mental and physical health outcomes in chronic populations in LMICs. The proposed trial will be the first RCT evaluating the real-world effectiveness of a task-shared collaborative team approach to caring for hypertensive patients with co-morbid depression in a LMIC.

### Trial status

At the time of submission (September 2016) the trial is in follow-up stage. Recruitment and baseline data collection began in April 2015, and will continue until the end of October 2016.

## Additional files

Additional file 1: Administration file_1 Jun 2017. (DOCX 40 kb)

Additional file 2: SPIRIT Checklist. (DOCX 23277 kb)

Additional file 3: PC101 pages. (DOCX 37 kb)

Additional file 4: Pre-consent process. (DOCX 492 kb)

Additional file 5: PRIME and COBALT consent forms. (PDF 7265 kb)

Additional file 6: Baseline questionnaire. (DOC 130 kb)

## Abbreviations

ART: Antiretroviral treatment
AUD: Alcohol use disorders
BP: Bojanala Platinum District
BREC: Biomedical Research Ethics Committee
CHD: Coronary heart disease
CHW: Community health worker
CobALT: Comorbid Affective Disorders AIDS/HIV and Long Term Health
CVD: Cardiovascular disease
DALYs: Disability-adjusted Life Years
DFID: Department for International Development
DKK: Dr. Kenneth Kaunda District
DSMB: Data and Safety Monitoring Board
DSM-IV: Diagnostic and Statistical Manual of Mental Disorders
EMERALD: Emerging Mental Health Systems in Low- and Middle-income Countries
HIC: High-income countries
HIV: Human immunodeficiency virus
HREC: Human Research Ethics Committee
ICC: Intraclass correlation coefficient
LMIC: Low- and middle-income countries
MRC: Medical Research Council
NCDs: Non-communicable diseases
NHANES: National Health and Nutrition Examination Survey
NIHR: National Institute for Health Research
PACIC: Patient Assessment of Care for Chronic Conditions
PC101: Primary Care 101
PHC: Primary health care
PHQ-9: Patient Health Questionnaire
PI: Principle investigator
PRIME: Programme for Improving Mental Health Care
PSS: Perceived Stress Scale
RCT: Randomised controlled trial
TB: Tuberculosis
UK: United Kingdom
UKZN: University of KwaZulu-Natal
VAS: Visual Analogue Scale
WBOT: Ward-based outreach team
WHO: World Health Organiszation
WHODAS: World Health Organiszation Disability Assessment Schedule
YLD: Years lived with disability
YLLs: Years of life lost
