# Supplementary material for: Collaborative care for the detection and management of depression among adults with hypertension in South Africa: study protocol for the PRIME-SA randomised controlled trial
Source: Trials. 2018 Mar 22;19:192. doi: 10.1186/s13063-018-2518-6 (PMC5863904; doi:10.1186/s13063-018-2518-6)
Supplement: Supplementary file 1 — Administration file_1 Jun 2017. (DOCX 40 kb) [file 13063_2018_2518_MOESM1_ESM.docx]

**COBALT Protocol: Additional file 1**

**Title and registration**

| **Section/ item** | **Details** |
| --- | --- |
| Title including acronym | Collaborative care for the detection and management of depression among adults receiving antiretroviral therapy in South Africa: study protocol for the CobALT randomised controlled trial |
| Trial Registration | ● ClinicalTrials.gov NCT02407691 Date of registration: 19/03/2015  ● Pan African Clinical Trials Registry PACTR201504001078347 Date of registration: 19/03/2015  ● South African National Clinical Trials Register (SANCTR) DOH-27-0515-5048 (NHREC number 4048 issued 21/04/2015) |
| Protocol version | 6 |
| Protocol date | 23 August 2016 |
| Funding | ● National Institute of Mental Health, grant 5R01MH100470-03 (2013-2018, trial itself)  ● Department for International Development (2011 to 2017, development and piloting of the intervention) |

**Roles and responsibilities**

| **Grouping** | **Responsibilities** | **Frequency** | **Membership** |
| --- | --- | --- | --- |
| Sponsors | Overall oversight and running of the trial | N/A | Shared by:  L Fairall ([Lara.Fairall@uct.ac.za](mailto:Lara.Fairall@uct.ac.za))  I Petersen ([PETERSENI@ukzn.ac.za](mailto:PETERSENI@ukzn.ac.za))  G Thornicroft ([graham.thornicroft@kcl.ac.uk](mailto:graham.thornicroft@kcl.ac.uk))  The responsibility between the three Co-principal investigators is divided as follows: L Fairall leads on data collection and analysis, I Petersen on implementation of the intervention, and G Thornicroft on relationship with the main funder. All three investigators are the guarantors for the trial. |
| Project Management Group | Review of intervention and data collection progress, compilation and submission of reports, consideration of any problems or methodological issues. | Monthly | L Fairall, I Petersen, G Thornicroft, N Folb, K Davies, B Zani, O Selohilwe, D Carter, D Georgeu-Pepper |
| Trial Co-ordinating Centre | Daily running of the intervention and data collection including weekly meetings to consider intervention process indicators, review of recruitment and follow-up targets, practical on-the-ground issues. | Daily, weekly | L Fairall, I Petersen, N Folb, B Zani, D Georgeu-Pepper, D Carter, M Mothaga, F Eshraghi, T Kathree, P Mothibedi, N Mntambo, R Petrus, O Selohilwe, V Ntjikelane |
| Trial Investigator Group | Protocol design, consideration of methodological issues, input into data analysis plan, interpretation and write up of results | Annual | L Fairall, I Petersen, B Zani, N Folb, D Georgeu-Pepper, O Selohilwe, A Bhana, C Lombard, M Bachmann, C Lund, J Hanass-Hancock, D Chisholm, P McCrone, S Carmona, T Gaziano, N Levitt, M Prince, S Saxena, E Susser, G Thornicroft |
| Data Safety and Monitoring Board (DSMB) |  | 6 monthly, ad hoc as needed | DSMB members: C Butler (Chair), H Weiss (Statistician), S Delany Moretlwe, C Orrell, J Joska  Research team members involved in the DSMB open sessions: L Fairall (PI), G Thornicroft (PI), I Petersen (PI), N Folb (project manager), B Zani (fieldwork coordinator)  Data Coordinating Centre representative for the closed sessions: L Fairall |
| Project Steering Committee |  | Annual | I Petersen, L Fairall, A Bhana, C Lund, M Bachmann, C van Deventer, M Freeman, S Phakathi, J de Beer, H Reichel,  S Asmall |

**Contributorship**

| **Activity** | **Contributors** |
| --- | --- |
| Study design | L Fairall, I Petersen, G Thornicroft, N Folb, A Bhana, C Lombard, M Bachmann, C Lund, T Gaziano, N Levitt, D Chisholm, P McCrone, M Prince, J Hanass-Hancock, S Saxena, E Susser, B Zhani, S Carmona |
| Application for funding | L Fairall, I Petersen, G Thornicroft, N Folb, A Bhana, C Lombard, M Bachmann, C Lund, T Gaziano, N Levitt, D Chisholm, P McCrone, M Prince, J Hanass-Hancock, S Saxena, E Susser |
| Intervention development, piloting and implementation | I Petersen, L Fairall, A Bhana, G Thornicroft, R Cornick, O Selohilwe, G Faris, D Georgeu-Pepper, D Carter, Petrus, N Mntambo, N Folb, T Kathree |
| Data collection planning | L Fairall, N Folb, I Petersen, A Bhana, G Thornicroft, V Timmerman, O Selohilwe, B Zani, D Georgeu-Pepper, D Carter |

**Details of Trial Registration with ClinicalTrials.gov** NCT02407691

| **Item** | **Detail** |
| --- | --- |
| ClinicalTrials.gov Identifier | NCT02407691 |
| First received | March 19, 2015 |
| Sponsor | University of Cape Town |
| Collaborators | King's College London  University of KwaZulu Natal |
| Purpose | With increasing access to antiretroviral therapy (ART) in South Africa, HIV has transitioned from a terminal illness to a long-term condition. It is likely to be accompanied by higher levels of disability and other chronic non-communicable diseases, resulting from the HIV itself, as well as adverse effects of medication. This requires an expansion of the purview of HIV care beyond direct HIV clinical care to also include a more comprehensive and integrated package of treatment and care for physical and mental conditions and their consequences. COBALT is a pragmatic cluster randomized controlled trial (RCT) in public sector primary care clinics in the North West Province of SA. It will assess mental health and HIV outcomes for depressed adults receiving ART by measuring the real-world effectiveness of a facility-based stepped care intervention combining depression case detection by non-physician clinicians with group counselling intervention delivered by lay-health workers. |
| Condition | HIV/AIDS Depression Chronic Diseases of Lifestyle |
| Intervention | Primary care 101 plus mental health Standard Primary Care 101 |
| Study type | Interventional |
| Study Design | Allocation: Randomized Intervention Model: Parallel Assignment Masking: Single Blind (Outcomes Assessor) Primary Purpose: Health Services Research |
| Official Title | Collaborative care for the detection and management of depression among adults receiving antiretroviral therapy in South Africa: a pragmatic cluster randomised controlled trial |
| Primary Outcome Measures | ● Viral load suppression (viral load value of <1000 RNA copies/ml) [ Time Frame: 12 months ] [ Designated as safety issue: Yes ]  ● PHQ-9 response (at least a 50% improvement in PHQ-9 score compared with baseline)  [ Time Frame: 6 months ] [ Designated as safety issue: Yes ] |
| Secondary Outcome Measures | ●PHQ-9 response at 12 months (at least a 50% improvement in PHQ-9 score compared with baseline) [ Time Frame: 12 months ] [ Designated as safety issue: Yes ]  ● Depression remission at 12 months [ Time Frame: 12 months ] [ Designated as safety issue: No ]  Score of less than 5 in PHQ-9  ● Mean PHQ-9 score at 6 and 12 months [ Time Frame: 6 and 12 months ] [ Designated as safety issue: No ]  Average score in the PHQ-9  ●Depression severity (categorised as mild, moderate, moderately severe or severe depression at 6 and 12 months)  Mild depression defined as a score of 5 to 9; moderate depression defined as a score of 10-14; Moderately severe depression defines as a core of 15-19; and Severe depression defined as a score of 20-27.  [ Time Frame: 6 and 12 months ] [ Designated as safety issue: Yes ]  ●Antidepressant treatment initiated or intensified  [ Time Frame: 12 months ] [ Designated as safety issue: No]  ●Counselling for depression by a clinic-based counsellor  [ Time Frame: 12 months ] [ Designated as safety issue: No]  ●Referral to specialist mental health worker/service  [ Time Frame: 12 months ] [ Designated as safety issue: No]  ● Viral load suppression at 12 months, defined as a viral load of <400 copies/ml  [ Time Frame: 12 months ] [ Designated as safety issue: No]  ● Virological failure defined as two viral load values >1000 copies/ml  [ Time Frame: 12 months ] [ Designated as safety issue: No ]  ● Change in viral load values over time  [ Time Frame: 12 months ] [ Designated as safety issue: No ]  ● Antiretroviral therapy programme retention [ Time Frame: 12 months ] [ Designated as safety issue: No ]  continuation in the ART programme  ● Appropriate maintenance on enrolment ART regimen and ART regimen switched to second line [ Time Frame: 12 months ] [ Designated as safety issue: No ]  ● Risk factors for cardiovascular diseases (blood pressure, weight, smoking status)  [Time Frame: 12 months ] [Designated as safety issue: No ]  ● Detection and treatment of other chronic diseases  [ Time Frame: 12 months ] [ Designated as safety issue: No ]  Identification of new cases of NCDs or risk factors of chronic diseases  ● Hospital admissions [ Time Frame: 12 months ] [ Designated as safety issue: Yes ]  number and duration of overnight hospital stays  ● Mortality [ Time Frame: 12 months ] [ Designated as safety issue: Yes ]  Mortality reported at loss to follow-up or through the South African Population register  ● Stress [ Time Frame: 12 months ] [ Designated as safety issue: No ]  Measured through the Perceived Stress Scale  ● Stigma [ Time Frame: 12 months ] [ Designated as safety issue: No ]  Measured through the 6-item internalised AIDS related stigma scale  ● ART adherence [ Time Frame: 12 months ] [ Designated as safety issue: No ]  30 day VAS self reported measure  ● Provision of integrated care from patient perspective will be assessed using the Patient Assessment of Care for Chronic Conditions (PACIC)  [ Time Frame: 12 months ] [ Designated as safety issue: No ] |
| Other Outcome Measures | ● Disability [ Time Frame: 12 months ] [ Designated as safety issue: No ]  Measured by WHODAS 2  ● Care Utilisation and resource use. [ Time Frame: 12 months ] [ Designated as safety issue: No ]  Measured by service use questionnaire  ●Productivity and economic outcomes  [ Time Frame: 12 months ] [ Designated as safety issue: No ]  Self-reported |
| Estimated Enrolment | 2000 |
| Study Start Date | April 2015 |
| Estimated Study Completion Date | December 2017 |
| Estimated Primary Completion Date | December 2017 (Final data collection date for primary outcome measure) |
| Arm: Experimental: | Assigned interventions:  Primary Care 101 Enhanced guideline  Primary Care 101 guideline with enhanced mental health |
| Arm: Active Comparator: | Assigned interventions:  Standard of care  Primary Care 101 standard version guideline |
| Ages Eligible for Study | 18 Years and older   (Adult, Senior) |
| Genders Eligible for Study | Both |
| Accepts Healthy Volunteers | No |
| Inclusion criteria | Clinics:  1) 40 nurse-led primary care clinics providing ART in the Dr Kenneth Kaunda and Bojonala districts of the North West Province, South Africa  Patients:   1. Age ≥ 18 years and 2. Receiving ART at the time of enrollment and 3. Depressive symptoms, as indicated by a total score of 9 or more on the PHQ-9 and 4. Planning to reside in the area for the next year and 5. Capable of actively engaging in an interviewer-administered questionnaire at the time of recruitment, six and twelve months later and 6. Written consent to participate in the study |
| Exclusion criteria | Clinics:  1) Clinics which participate in the formative research and piloting of the intervention  Patients:  1) Inability to meet the above inclusion criteria |

**PRIME Protocol: File 1**

**Title and registration**

| **Section/ item** | **Details** |
| --- | --- |
| Title including acronym | Collaborative care for the detection and management of depression among adults with hypertension in South Africa: study protocol for the PRIME-SA randomised controlled trial |
| Trial Registration | ● ClinicalTrials.gov: NCT02425124. Date of registration 22/04/2015  ● South African National Clinical Trials Register: SANCTR DOH-27-0916-5051 (NHREC number 4051 issued 09/04/15) |
| Ethical approval | Biomedical Research Ethics Administration: BFC 049/15 |
| Protocol version | 5 |
| Protocol date | 11 May 2016 |
| Funding | ● UK Department for International Development |

**Roles and responsibilities**

| **Grouping** | **Responsibilities** | **Frequency** | **Membership** |
| --- | --- | --- | --- |
| Sponsors | Overall oversight and running of the trial | N/A | Shared by:  Fairall ([Lara.Fairall@uct.ac.za](mailto:Lara.Fairall@uct.ac.za))  Petersen ([PETERSENI@ukzn.ac.za](mailto:PETERSENI@ukzn.ac.za))  Thornicroft (Graham. Thornicroft @kcl.ac.uk)  The responsibility between the two Co-principal investigators is divided as follows: Fairall leads on data collection and analysis, Petersen on implementation of the intervention. Thornicroft provides overall oversight. All 3 Investigators are guarantors for the trial. |
| Project Management Group | Review of intervention and data collection progress, compilation and submission of reports, consideration of any problems or methodological issues. | Monthly | Fairall, Petersen, Thornicroft, Folb, Davies, Zani, Selohilwe, Carter, Georgeu-Pepper |
| Trial Co-ordinating Centre | Daily running of the intervention and data collection including weekly meetings to consider intervention process indicators, review of recruitment and follow-up targets, practical on-the-ground issues. | Daily, weekly | Fairall, Petersen, Folb, Zani, Georgeu-Pepper, Carter, Mothaga, Eshraghi, Kathree, Mothibedi, Mntambo, Petrus, Selohilwe, Ntjikelane |
| Trial Investigator Group | Protocol design, consideration of methodological issues, input into data analysis plan, interpretation and write up of results | Annual | Fairall, Petersen, Thornicroft, Folb, Zani, Selohilwe, Bhana, Lombard, Bachmann, Lund, Gaziano, Levitt, Chisholm, McCrone, Prince, Georgeu-Pepper |
| Data Safety and Monitoring Board (DSMB) |  | 6 monthly, ad hoc as needed | DSMB members: Butler (Chair), Weiss (Statistician), Delany Moretlwe, Orrell, Joska    Research team members involved in the DSMB open sessions: Fairall (PI), Thornicroft (PI), Petersen (PI), Folb (project manager)  Data Coordinating Centre representative for the closed sessions: Fairall |
| Project Steering Committee |  | Annual | Petersen, Fairall, Bhana, Lund, Bachmann, van Deventer, Freeman, Phakathi, de Beer, Reichel,  Asmall |

**Contributorship**

| **Activity** | **Contributors** |
| --- | --- |
| Study conceptualization | I Petersen, L Fairall, A Bhana, G Thornicroft, C Lund |
| Study design | I Petersen, L Fairall, G Thornicroft, N Folb, A Bhana, C Lombard, M Bachmann, C Lund, T Gaziano, N Levitt, D Chisholm, P McCrone, M Prince, B Zani |
| Application for funding | I Petersen, L Fairall, A Bhana, C Lund, T Kathree |
| Intervention development , piloting and implementation | I Petersen, L Fairall, A Bhana, G Thornicroft, R Cornick, O Selohilwe, G Faris, D Georgeu-Pepper, D Carter, R Petrus, N Mntambo, N Folb, T Kathree |
| Data collection planning | L Fairall, N Folb, I Petersen, A Bhana, G Thornicroft, V Timmerman, O Selohilwe, B Zani , D Georgeu-Pepper, D Carter |

**Details of Trial Registration with ClinicalTrials.gov** NCT02425124 Date of registration 22/04/2015

| **Item** | **Detail** |
| --- | --- |
| ClinicalTrials.gov Identifier | NCT02425124 |
| First received | 22/04/2015 |
| Sponsor | University of KwaZulu-Natal  InQubate  Research Office  Govan Mbeki Centre  Westville Campus  Tel: +27 (31) 260 7291  Fax: +27 (31) 260 2384 |
| Collaborators | King's College London  University of Cape Town |
| Purpose | A pragmatic cluster randomized controlled trial (RCT) in 20 public sector primary care clinics in the Dr Kenneth Kaunda district of the North West Province of South Africa to assess mental health and health outcomes for depressed adults receiving hypertensive treatment by measuring the real-world effectiveness of a facility-based stepped care intervention combining stress and depression case detection and management by non-physician  clinicians and referral pathways for anti-depressant medication and/or group/individual counselling delivered by lay-health workers for patients with depression. The control condition is enhanced usual primary health  care where non-physician clinicians have been equipped with the basic skills to identify stress and depression/anxiety but with limited access to doctors authorized to prescribe antidepressant medication, and with no specific  psychosocial interventions. |
| Condition | Depression Hypertension |
| Intervention | Primary care 101 plus mental health Standard Primary Care 101 |
| Study type | Interventional |
| Study Design | Allocation: Randomized Intervention Model: Parallel Assignment Masking: Single Blind (Outcomes Assessor) Primary Purpose: Health Services Research |
| Official Title | Strengthening Mental Health Care in Chronic Care Patients With Hypertension. A Cluster Randomised Control Trial |
| Primary Outcome Measures | Depression  ● Reduced depressive symptoms (measured by Patient Health Questionnaire 9 (PHQ-9) score) [ Time Frame: 6 months  [ Designated as safety issue: Yes ]  50% reduction in the PHQ-9 score |
| Secondary Outcome Measures | Depression  ● Response at 12 months (measured by PHQ-9 score) [ Time Frame: 12 months] [ Designated as safety issue: Yes ]  50% reduction in the PHQ-9 score  ● Remission at 12 months [ Time Frame: 12 months] [ Designated as safety issue: No ]  Score of less than 5 in PHQ-9  ● Mean PHQ-9 score at 6 and 12 months [ Time Frame: 6 and 12 months ] [ Designated as safety issue: No ]  Average score in the PHQ-9  ●Blood pressure control as measured by trained fieldworker [Time Frame: 12 Months] [Designated as safety issue: Yes]  • Disability measured using the Manual for WHO Disability Schedule WHODAS 2 .0 [Time Frame: Baseline; 12 Months] [Designated as safety issue: No] |
| Other Outcome Measures | **Secondary mental health outcomes**   - Perceived stress symptoms assessed using the Perceived Stress Scale [Time Frame: Baseline; 12 Months] [Designated as safety issue: No] - Antidepressant treatment initiated or intensified - Counselling by clinic-based counsellor - Referral to specialist mental health worker/ service   **Secondary Hypertensive Outcomes**   - Retention in care   **Integrated care outcomes**   - Cardiovascular risk factors: blood pressure, weight, body mass index, waist circumference - Diagnosis of other comorbid illnesses - Quality of chronic illness care received: Patient Assessment of Care for Chronic Conditions (PACIC)   **Health economic outcomes**   - Healthcare utilization - Productivity and economic outcomes |
| Safety measurements | - Hospitalisation - All-cause mortality |
| Estimated Enrolment | 1000 |
| Study Start Date | April 2015 |
| Estimated Study Completion Date | December 2016 |
| Estimated Primary Completion Date | November 2016 (Final data collection date for primary outcome measure) |
| Arm: Experimental: | Facility-based stepped care intervention combining stress and depression case detection and management by non-physician  clinicians and referral pathways for anti-depressant medication and/or group/individual counselling delivered by lay-health workers for patients with depression  Assigned interventions:  Primary Care 101 Enhanced guideline  Primary Care 101 guideline with enhanced mental health |
| Arm: Active Comparator: | Enhanced usual primary health  care where non-physician clinicians have been equipped with the basic skills to identify stress and depression/anxiety but with limited access to doctors authorized to prescribe antidepressant medication, and with no specific  psychosocial interventions.  Assigned interventions:  Standard of care  Primary Care 101 standard version guideline |
| Ages Eligible for Study | 18 Years and older   (Adult, Senior) |
| Genders Eligible for Study | Both |
| Accepts Healthy Volunteers | No |
| Inclusion criteria | Clinics:  1) 20 largest primary care clinics providing ART in the Dr Kenneth Kaunda providing chronic care of the North West Province, South Africa  Patients:   1. Age ≥ 18 years and 2. Receiving hypertensive treatment at the time of enrolment and 3. Depressive symptoms, as indicated by a total score of 9 or more on the PHQ-9 and 4. Planning to reside in the area for the next year and 5. Capable of actively engaging in an interviewer-administered questionnaire at the time of recruitment, six and twelve months later and 6. Written consent to participate in the study |
| Exclusion criteria | Clinics:  1) Clinics that do not provide Integrated Chronic Disease Management  • Small (<10 000 attendances/ year)  • Mobile or satellite  • Participated in piloting of intervention & data collection  Patients:  1) Inability to meet the above inclusion criteria |
| Contacts | Contact: Inge Petersen +27312601709  email: [peterseni@ukzn.ac.za](mailto:peterseni@ukzn.ac.za)  University of KwaZulu-Natal  Private Bag X54001  Westville  3630  Attention: Prof Inge Petersen  Centre for Rural Health  Room 402 George Campbell Building (code 62)  Howard College Campus    Contact: Lara Fairall, PhD +27214066920 [lara.fairall@uct.ac.za](mailto:lara.fairall@uct.ac.za)  Attention: Dr Lara Fairall  Knowledge Translation Unit University of Cape Town Lung Institute PO Box 34560 Groote Schuur 7937 South Africa |
| Sponsors and collaborators | University of Cape Town  King's College London  University of KwaZulu-Natal |
| Investigators | Principal Investigator: Inge Petersen, PhD University of KwaZulu-Natal, Durban, South Africa  Principal Investigator: Lara Fairall, PhD University of Cape Town, Cape Town, South Africa  Principal investigator: Graham Thornicroft, PhD  King’s College London, London, England |
| Additional information | Responsible Party: Inge Petersen www.ukzn.ac.za, University of KwaZulu-Natal  ClinicalTrials.gov Identifier: NCT02425124  Other Study ID Numbers: DOH-27-0916-5051  Study First Received: 24 March 2015  Last Updated: 09 September, 2016  Health Authority: South Africa: Data and Safety Monitoring Board  Funder: United Kingdom: Department of International Development  Additional relevant MeSH terms:  Chronic Disease  Disease Attributes  Pathologic Processes |
