# Supplementary material for: Collaborative care for the detection and management of depression among adults with hypertension in South Africa: study protocol for the PRIME-SA randomised controlled trial
Source: Trials. 2018 Mar 22;19:192. doi: 10.1186/s13063-018-2518-6 (PMC5863904; doi:10.1186/s13063-018-2518-6)
Supplement: Supplementary file 3 — PC101 pages. (DOCX 37 kb) [file 13063_2018_2518_MOESM3_ESM.docx]

**Additional file 3: Pre-consent processes including management of patients who express end-of-life thoughts**

**Definitions**

**Recruiter:** A fieldworker who is responsible for identifying potential participants from the waiting room, and referring them to the interviewer for screening for enrolment in the trial.

**Interviewer:** A fieldworker who is responsible for detailed screening of potential participants, completing the informed written consent process with patients, completing interviews, and care of patients who express end-of-life thoughts during the screening process.

**End-of-life thoughts:** For the purpose of this SOP, end-of-life thoughts are defined as answering ‘Yes’ to the following question which is the last question of the Patient Health Questionnaire-9: “Over the last 2 weeks, how often have you been bothered by any of the following problems - thoughts that you would be better off dead or of hurting yourself in some way?”

**Step-by-step procedure to be followed:**

1. In the waiting room, the Recruiter gives the following information to the group of patients assembled there. A suggested script is provided:

*“My name is xx and I am employed by the University of Cape Town to work on two studies investigating whether counselling helps improve health outcomes for people with chronic conditions. We are looking for clinic patients over the age of 18 years with certain chronic diseases to take part. These studies will help us to improve the treatment chronic patients receive at clinics in South Africa. The studies will involve being interviewed today and twice more, in 6 months’ time and in 12 months’ time. For now, we are asking you to agree to being asked three short questions to see if you might be suitable for the studies. We will ask you some more detailed questions to determine whether you qualify to take part in either or both of the studies. Should you qualify we will also give you more detailed information about the full studies so that you can decide whether or not to take part. Your care at the clinic will not be affected in any way if you choose not to take part. But we hope you will take part. I will be based [state where the recruiter will be based e.g. in the waiting room or interview room] so please come and tell me if you are interested in taking part in these important studies.”*

Note: no mention of depression or HIV which may be stigmatising in a group context, and in the case of the former, may bias the sample if potential participants think they already have to have a diagnosis of depression to qualify.

1. The Recruiter engages with interested patients, one on one, either in a quiet corner of the waiting room or preferably in a private space close to where the interviews are being conducted. He/ she asks the interested patient the following 3 questions:

*“Thank you for your interest in the study. We are looking for patients on high blood pressure treatment and/or antiretrovirals who plan to stay in the local area for the duration of the study. Can I therefore ask:*

1. *Are you currently taking medicine for high blood pressure (hypertension or high blood)?*
2. *Are you currently taking ARVs (antiretrovirals)?*

If yes to [i] and/or [ii] ask:

1. *Are you planning to stay in the area for the next year?*

If yes to [i] and/or [ii] AND yes to [iii]:

*“It looks like you are suitable to be screened for participation in the study. My colleague who is completing the full interviews will screen you using some questions. Are you still interested in taking part?”*

If no to these questions, thank the patient for their time, remind them their care at the clinic will not be affected because they didn’t qualify for the study and wish them well.

1. Recruiter arranges for Interviewer to screen potential participant.
2. The Interviewer now engages with the potential participant in a private space. He/ she starts by asking in what language the potential participant would like to be interviewed, selecting the appropriate interview option on the handheld device, and capturing the date and clinic in which the screen is being done. No patient identifiers are captured.
3. (Built into electronic questionnaire.) The interviewer then proceed with the electronic questionnaire which includes the following opt-out consent to be screened using PHQ-9:

*“As you have heard from my assistant we are conducting two studies to evaluate a programme and we are looking for people with certain criteria to take part. I would like to start by asking you a few questions to see whether you qualify to take part in either or both of the studies. Some of these questions are about your psychological well-being and may be sensitive. You are free to stop the interview at any time and this will not affect your usual care at the clinic. Are you willing to continue with the questionnaire?”*

If no, Interviewer to read: ‘We would like to thank you for your time today and we wish you well.’

If yes, continue.

1. (Built into electronic questionnaire.) Continue electronic questionnaire to PHQ-9 and three questions on whether or not the patient is on antihypertensives or antidepressants and planning to stay in the vicinity of the clinic for the study period.

If patient answers that they have had end-of-life thoughts in response to PHQ-9 Question 9 (i.e. 1-7 days, 8-11 days, 12-14 days), repeat that question (built into questionnaire programme).

If 1-7 days, Interviewer to read: *‘At the end of the interview I will give you a leaflet with some contact details for people who will be able to help you with how you are feeling.’*

If 7 days or more, Interviewer to read: *‘I am concerned about how you are feeling and would like to ask a nurse to see you at the end of this interview.’*

If not eligible: Skip to end of questionnaire.

If eligible, continue.

Note: No confidential information (full names, date of birth, contact details) may be recorded for patients who have not yet provided written informed consent.

1. Interviewer to go through written consent process for eligible patients. Interviewer goes through patient information sheets for one or both studies, explaining study procedures, potential risks and benefits and asks patient whether or not they are still willing to participate.

If yes, the Interviewer continues with the interview, now capturing patients’ confidential information.

If no, continue to 8.

1. (Built into electronic questionnaire.) Interviewer to review patient/ participant’s status in terms of end-of-life thoughts, as prompted by computer.

If the patient confirmed end-of-life thoughts further actions to be guided by frequency of those thoughts.

Less than 7 days in the last 2 weeks: Provide patient with written material on depression and suicide prevention.

7 days or more in the last 2 weeks: Complete urgent referral form, and accompany patient to clinic staff. Do not leave patient unattended. Wait until patient has been handed over to clinic staff.

**Figure: Overview of pre-consent processes and management of patients who express end-of-life thoughts**

Recruiter asks patients who come forward (one on one):

On ARVs and/or antihypertensive? *and*

Staying in the vicinity of the clinic for the next

Recruiter gives patients assembled in waiting room information on study and invites them to participate

Interviewer in private space starts electronic questionnaire for complete screen.

Opt-out consent (verbal) for PHQ-9 questions

Consent granted

Thank you and goodbye

Thank you and goodbye

Consent refused

Complete interview

Thank you and goodbye

Consent granted

Complete interview

Consent refused

End-of-life thoughts present and confirmed (question repeated)

Less than 7 days in last 2 weeks

7 days or more in last 2 weeks

Written information given

Referral and walked across to clinic staff

Eligible

“No” to end-of-life thoughts

Eligible

“Yes” to end-of-life thoughts

Not eligible

“Yes” to end-of-life thoughts

Not eligible

“No” to end-of-life thoughts

Thank you and goodbye

*No*

*Opts out*
